# Twice-spraying plant growth regulator EDAH stage-regulates maize plant morphology: a novel strategy for enhancing stalk lodging resistance

**DOI:** 10.3389/fpls.2026.1853755

**Published:** 2026-06-23

**Authors:** Fan Liu, Chaode Liang, Zongxiang Guo, Pijiang Yin, Chengcheng Lyu, Ye Tao, Dongju Feng, Xinglong Wang, Fanlei Kong, Jichao Yuan

**Affiliations:** 1 College of Agronomy, Sichuan Agricultural University, Key Laboratory of Crop Ecophysiology and Farming System in Southwest China, Ministry of Agriculture, Crop Ecophysiology and Cultivation Key Laboratory of Sichuan Province, Chengdu, China; 2 College of life Science and Agri-forestry, Southwest University of Science and Technology, Mianyang, China

**Keywords:** EDAH, internode length, maize, plant morphology, Stalk lodging resistance

## Abstract

Spraying the plant growth regulator EDAH (27% ethephon and 3% diethyl aminoethyl hexanoate) to enhance maize lodging resistance is a widely accepted practice in maize cultivation. In 2021–2022, single applications of EDAH at V6 (6 expanded leaves) and V12 (12 expanded leaves) were ineffective in preventing lodging (lodging rate, 21.76%–29.00%), though they did shorten internode lengths at the basal and middle-upper sections, respectively. Consequently, a twice-spraying regimen of EDAH at V6 and V12 was adopted in 2024–2025 to modify plant morphology. The effects of this twice-spraying were evaluated by determining grain yield and indicators related to stalk lodging resistance. Twice-spraying EDAH significantly improved stalk lodging resistance, reducing the lodging rate by 64.12%–79.28% and increasing grain yield by 2.26%–7.96%. This treatment simultaneously shortened the basal second to the fifth and middle-upper seventh to the eleventh internode lengths (by 21.07%–33.54% and 20.73%–32.76%, respectively), leading to significant reductions in plant height (20.67%) and ear height (23.90%), ultimately lowering the gravity height (19.06%). Leaf angles were also reduced, particularly at high planting densities. The primary mechanism by which twice-spraying EDAH reduced lodging rates was the optimization of plant morphology. The gravity height contributed 40.00% to lodging susceptibility. Twice-spraying EDAH enables staged regulation of maize stalk development, offering new insights and a production strategy for establishing dense-planted, stalk lodging-resistant, and consistently high-yielding maize populations.

## 1 Introduction

Maize (*Zea mays* L.) lodging is a major problem that limits high and stable yields (Li et al., 2021; Huang et al., 2025) and increases time and labor costs during mechanical harvesting (Wang et al., 2021). Its widespread occurrence has been extensively reported (Huang et al., 2021; Ren et al., 2024). Lodging damages the canopy structure (Robertson et al., 2015), which reduces dry matter accumulation, impedes water and nutrient transport, and can even decrease the number of effective ears, thereby lowering grain yield (Li et al., 2021; Cui et al., 2022). Maize lodging occurs either as stalk lodging, caused by the breaking or bending of internodes below the ear, or root lodging, resulting from the loss of integrity in the root-soil anchoring system (Stubbs et al., 2020; Liu et al., 2023). Stalk lodging accounts for over 60% of lodging in densely planted maize (Wang et al., 2020; Li et al., 2021), leading to the breakage of vascular tissue and greater grain yield losses (Robertson et al., 2015; Xue et al., 2016; Liu et al., 2021). Therefore, enhancing stalk lodging resistance can improve both grain yield and production efficiency.

Stalk lodging is determined by external forces (wind speed, pests, disease, and canopy airflow) and stalk lodging resistance (Stubbs et al., 2020; Kong et al., 2024). Due to the uncertainty of external factors, researchers have increasingly focused on traits related to stalk lodging resistance (Wang et al., 2020; Liu et al., 2023; Kong et al., 2024). Basal internodes support the aboveground portion, serving as the site of stress deformation and lodging occurrence (Liu et al., 2023). Their mechanical strengths and associated traits are closely linked to stalk lodging (Zhan et al., 2022; Kong et al., 2024). Reduced plant height, ear height, gravity height, and aboveground fresh weight decrease the bending moment exerted at the basal internode (Robertson et al., 2017; Zhao and Zhou, 2022). Additionally, more upright and smaller upper leaves reduce wind drag force (Tian et al., 2019; Zhou et al., 2024). These factors are beneficial in decreasing the field lodging rate.

Enhancing stalk lodging resistance through breeding hybrids and developing management strategies has attracted significant attention. The application of dwarf or semi-dwarf genes in wheat and rice has led to dramatic increases in planting density and grain yield (Yi et al., 2019; Ren et al., 2024; Zhou et al., 2024). In contrast, maize-related mutants and genes significantly reduce grain yield and have not been widely adopted (Nelissen et al., 2020; Han et al., 2022; Sun et al., 2024). Breeding hybrids with excellent lodging resistance and high grain yield remains challenging in the short term (Nelissen et al., 2020). Therefore, spraying plant growth regulators as an effective strategy to balance high grain yield with a low stalk lodging rate is widely used (Ren et al., 2023; Wang et al., 2024; Hong et al., 2025).

Plant growth regulators regulate maize stalk and ear growth and development by adjusting endogenous hormones (Liu et al., 2021; Xu et al., 2023). Ethephon (ETH) is commonly used to enhance stalk lodging resistance by regulating stalk elongation, thereby reshaping plant architecture (Ren et al., 2022; Xie et al., 2023), but it also reduces plant growth rate and leaf area index (Geng et al., 2022; Ren et al., 2022). Spraying ETH during the critical period of ear development decreased kernel number, thus lowering grain yield (Zhang et al., 2019; Geng et al., 2022; Huang et al., 2025), especially during V8-V12 (Zhao et al., 2022a). Diethyl aminoethyl hexanoate (DA-6) could enhance maize leaf photosynthetic capacity and accelerate ear development during the later reproductive stage, especially after tasseling, thereby increasing grain yield (Wang et al., 2024). The combined application of ETH and DA-6 (EDAH) could also effectively enhance maize stalk lodging resistance by optimizing plant morphology (Xu et al., 2017; Hong et al., 2025). Additionally, spraying EDAH promoted ear differentiation and enhanced the grain-filling rate to increase grain yield (Wang et al., 2024). However, single-spraying plant growth regulator is not completely effective in optimizing plant morphology and preventing maize lodging. Farmers in certain regions have been observed adopting twice-spraying EDAH in maize production, with the first at V6 (stalks begin to elongate) and the second at V12 (plant rapid growth). However, there is currently no systematic research on this noteworthy production technology.

It is presumed that twice-spraying EDAH could significantly enhance maize stalk lodging resistance and maintain high grain yield. The objectives were (1) to evaluate the responses of grain yield and stalk lodging resistance to the twice-spraying EDAH; (2) to propose a model for maize stalk dwarfing through the twice-spraying EDAH; and (3) to achieve an increase in grain yield under higher planting density. This study provides new insight and a technical strategy for spraying plant growth regulators to reduce maize stalk lodging in maize planting systems.

## 2 Materials and methods

### 2.1 Experiment site, design and field management

Field experiments were conducted at the Zhongjiang Experimental Station of Sichuan Agricultural University, Sichuan China (31.03°N, 104.68°E) in the 2021–2022 and 2024–2025 growing seasons. The site experiences a humid subtropical monsoon climate, with concentrated rain and strong winds frequently during the late maize growth stage. Daily meteorological data from sowing to harvest were obtained from the Zhongjiang Meteorological Station, including precipitation, maximum and average wind speed, maximum, minimum and mean temperatures, and sunshine hours, as presented in **Supplementary Figure 1**.

The experiments were arranged in a randomized block design. In 2021-2022, three plant growth regulator treatments were established: control with water (CK), single-spraying EDAH at V6 (SV6), and single-spraying EDAH at V12 (SV12). However, single-spraying EDAH did not effectively prevent lodging. Based on the 2021–2022 results, a novel spraying technology was implemented in 2024–2025, involving twice-spraying EDAH at V6 and V12 (TS) to replace single-spraying. The plant growth regulator Aifeng (Sichuan Guoguang Agrochemical Co., Ltd., Chengdu, Sichuan, China), containing 27% ETH and 3% DA-6 as active ingredients, was applied. The solution concentration was 6 mL L^-1^, and the dose was 750 L ha^-1^. Aifeng and water were sprayed by a backpack electric sprayer at 16:00–18:00. Specific spraying dates are provided in **Supplementary Figure 1**. Two maize hybrids with contrasting lodging resistance, Chengdan30 (CD30, lodging-resistant) and Zhenghong505 (ZH505, lodging-susceptible), both widely promoted locally and having essentially the same growth durations (Liu et al., 2025), were used. In 2021–2022, only ZH505 was used, while in 2024–2025, both CD30 and ZH505 were used. Planting densities were 4.5 × 10^4^ plants ha^−1^ (D_4.5_) in 2021–2022 and 5.25 × 10^4^ plants ha^−1^ (D_5.25_) in 2024. In 2025, in addition to D_5.25_, a planting density treatment of 8.25 × 10^4^ plants ha^−1^ (D_8.25_) was added to achieve higher grain yields and verify the efficacy of twice-spraying EDAH at high planting density. Each treatment was replicated three times, with each plot area being 31.5 m^2^ (4.5 m × 7 m) and configured with 100 cm + 50 cm row spacing. Basal fertilization before sowing provided 120 kg N ha^-1^ (urea), 72 kg P_2_O_5_ ha^-1^ (diammonium phosphate), and 90 kg K_2_O ha^-1^ (potassium sulphate), placed in furrows parallel to the rows. A supplemental application of 120 kg N ha^-1^ was side-dressed in holes dug near plants at the V12 stage. Efficient and timely agronomic management practices were implemented throughout the growing period to minimize interference from weeds, diseases, and pests. The sowing schedules and reproductive processes in 2021–2022 and 2024–2025 are provided in **Supplementary Table 1**.

### 2.2 Sampling and measurement methods

At silking, five representative plants per plot with uniform silking were tagged. Leaf angle, plant and internode morphological traits, the third internode mechanical strengths, and matter constituents were measured 20 days after silking. Lodging rate was assessed before harvest at physiological maturity, after which grain yield and its components were determined. In 2021–2022, only plant and basal internode morphologies, mechanical strengths, lodging rate, and grain yield were measured.

#### 2.2.1 Leaf angles and plant morphological traits

At 20 days after silking, the leaf angle (the angle between the leaf midrib and stalk) (Liu et al., 2025) of the ear leaf, the third leaf above, and the third leaf below the ear was measured in the field using a protractor on five marked plants per plot. The tagged plants were then cut at ground level. A ruler was used to measure plant height (PH), ear height (EH), and gravity height (GH). PH was defined as the length from the first basal node to the top of the tassel, EH as the length from the first basal node to the node bearing the ear, and GH as the length from the first basal node to the balance point (Kong et al., 2024). The ear height coefficient (EHC) and gravity height coefficient (GHC) were calculated using (**Equations 1**, **2**), respectively (Kong et al., 2024).

(1) $$\text{EHC }(\%)=\frac{EH}{PH}\times 100$$

(2) $$\text{GHC }(\%)=\frac{GH}{PH}\times 100$$

#### 2.2.2 Internode morphological traits

After measuring plant morphological traits and removing sheaths, internode morphological traits were measured. First, the length of all internodes was measured with a measuring tape. The minor and major diameters of the first to the seventh basal internodes were determined at the internode midpoint using a Vernier caliper, and their mean was used as the internode diameter (Liu et al., 2023). The internode length-diameter ratio (LDR) was calculated using the following formula:

(3) $$\text{LDR }(\text{cm }cm^{-1})=\frac{internode\;length}{internode\;diameter}$$

#### 2.2.3 Internode mechanical strength and matter constituent

The rind penetration strength (RPS) and bending strength (BS) of the third basal internode were determined after measuring internode morphology using an AWOS-SL04 stalk strength tester (Shijiazhuang Aiwoshi Technology Co., Ltd., Shijiazhuang, Hebei, China) (Kong et al., 2024). During RPS measurement, a needle probe (1 cm long, 1 mm^2^ cross-sectional area) penetrated only the maize internode rind. For BS, a U-shaped probe broke the entire stalk. These probes were operated by the same researcher at a constant, slow speed to penetrate or break internodes, and the peak values displayed on the monitor were recorded.

The third basal internodes were cut from plants and weighed to determine fresh weight. The remaining plant parts were separated into leaf, stalk + sheath, ear, and tassel + bract. These samples were first dried at 105°C for 30 min and then at 80 °C until a constant weight was achieved. Internode moisture content (MC) and internode plumpness (IP) were calculated using (**Equations 4**, **5**), respectively (Liu et al., 2023). The middle 3–4 cm of dried internode samples were cut, crushed, and sifted through a 0.5-mm sieve. The unscreened portion was analyzed for lignin content (LC), cellulose content (CC), and hemicellulose content (HC) using the Van–Soest method (Gong et al., 2021). The portion passing through the screen was analyzed for soluble sugar content (SC) and amylum content (AC) via anthrone colorimetry (Wu et al., 2019).

(4) $$\text{MC }(\%)=(1-\frac{internode\;dry\;mass}{internode\;fresh\;weight})\times 100$$

(5) $$\text{IP }({\text{g cm}}^{-1})=\frac{internode\;dry\;mass}{internode\;length}$$

#### 2.2.4 Stalk lodging resistance index, lodging rate, and grain yield

Stalk lodging resistance index (LI) was calculated using (**Equation 6**) (Kong et al., 2024). Lodged plants were counted and classified into stalk lodging (i.e., stalk broken at or below the ear-bearing node) and root lodging (i.e., stalk unbroken but leaning more than 45°from vertical) at physiological maturity (Hong et al., 2025).The stalk lodging rate, root lodging rate, and total lodging rate were then calculated using (**Equations 7**–**9**). The middle four unsampled rows of each plot were used to determine maize grain yield and its components. Before harvesting, the number of effective ears was counted, and 20 consecutive ears were selected to examine grains per ear and 100-grain weight. Grain yield was standardized at 14.0% moisture content.

(6) $$\text{Stalk lodging resistance index}=\frac{BS\;of\;the\;3rd\;internode}{GH}$$

(7) $$\text{Stalk lodging rate }(\%)=\frac{number\;of\;stalk\;lodged\;plants}{total\;number\;of\;plants}\times 100$$

(8) $$\text{Root lodging rate }(\%)=\frac{number\;of\;root\;lodged\;plants}{total\;number\;of\;plants}\times 100$$

(9) Lodging rate (%)=stalk lodging rate +root lodging rate

### 2.3 Statistical analysis

Statistical analysis was performed using the SPSS software by IBM Inc. (version 27.0, SPSS Inc., Armonk, NY, USA). Differences among the means were evaluated using the least significant difference (LSD) test. GraphPad Prism (version 9.4.1, GraphPad Software, Inc., Boston, MA, USA) and Photoshop 2025 (version 2025, Adobe, San Jose, CA, USA) were used to produce the figures. The correlation-path coefficient method was used to calculate the contributions of grain yield components to grain yield (Wei et al., 2025). Redundancy analysis (RDA) was performed using CANOCO 5.0 to examine relationships between plant traits related to stalk lodging resistance and lodging rate (Kong et al., 2024).

## 3 Results

### 3.1 Grain yield, lodging rate, and stalk lodging resistance index

**Figure 1:**
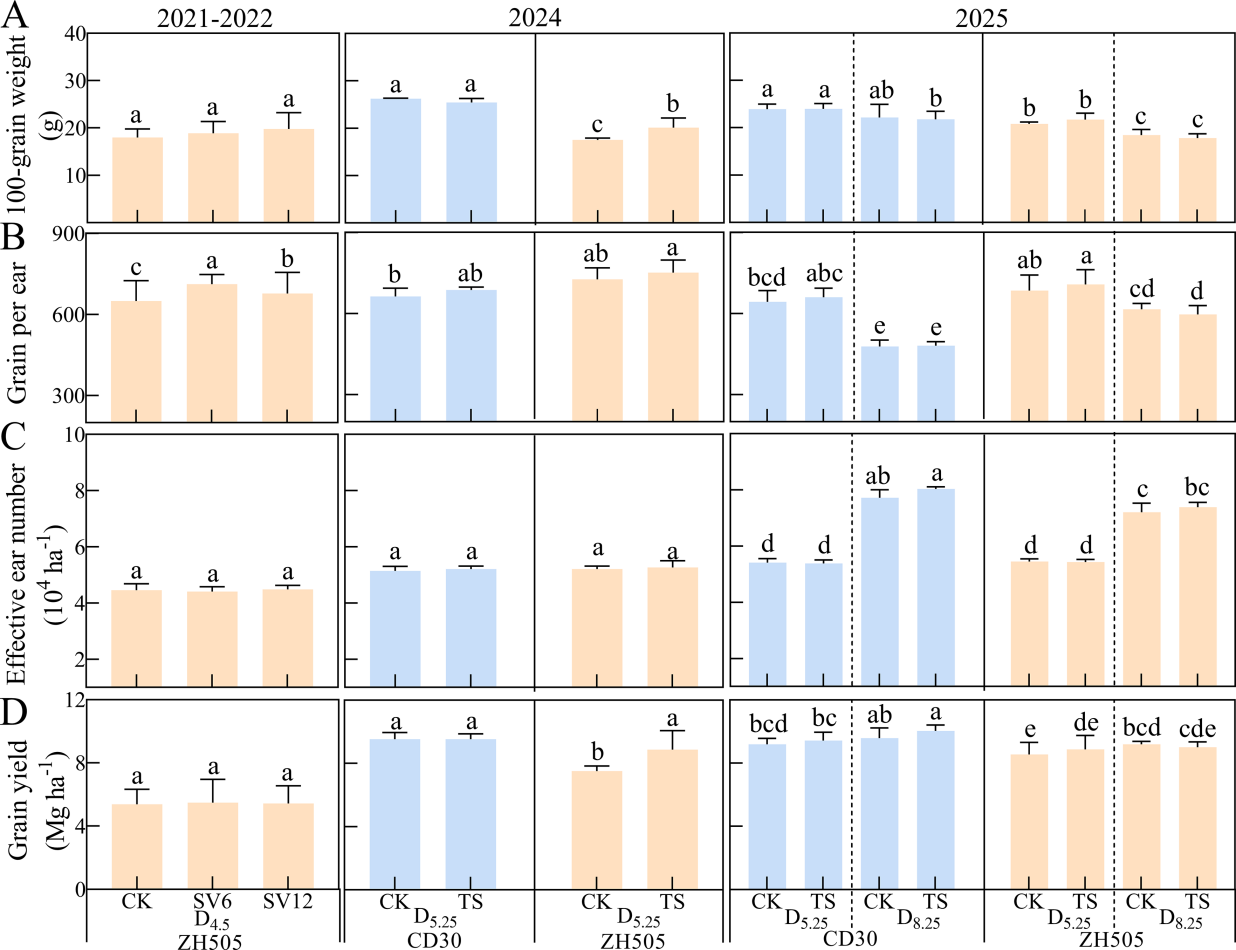
Maize yield components **(A–C)** and grain yield **(D)** in 2021–2022 and 2024–2025. Vertical bars display the standard errors. Different lowercase letters indicate significant differences (*P < 0.05*) between different treatments, as determined by the LSD multiple range test. CK, control treatment; SV6, spraying EDAH at V6; SV12, spraying EDAH at V12; TS, twice-spraying EDAH; D_4.5_, planting density of 4.5 × 10^4^ plants ha^-1^; D_5.25_, planting density of 5.25 × 10^4^ plants ha^-1^; D_8.25_, planting density of 8.25 × 10^4^ plants ha^-1^.

**Figure 2:**
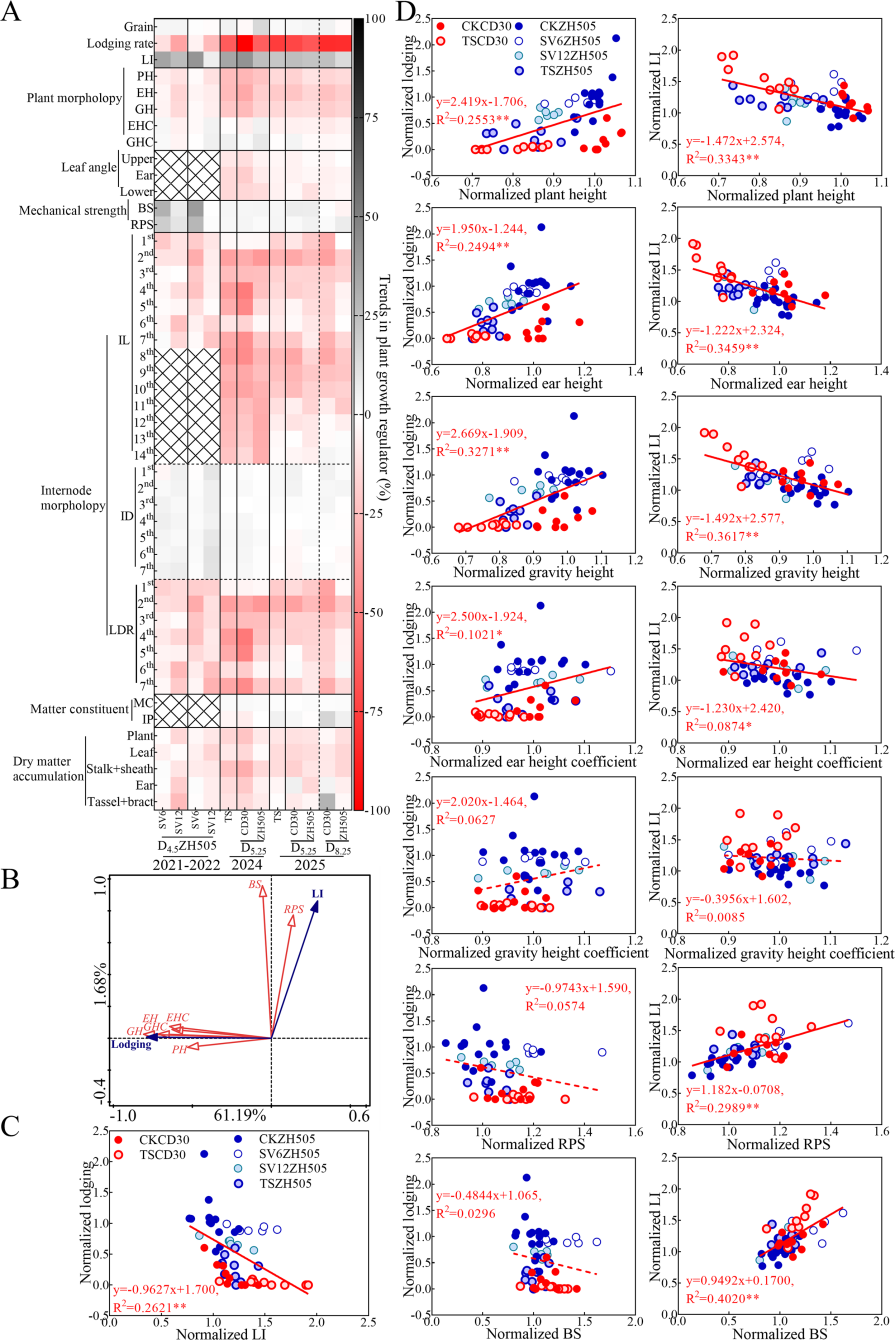
**(A)** Trend in stalk lodging resistance indicators, grain yield, and dry matter accumulation under different spraying methods of the plant growth regulator EDAH. **(B)** Redundancy analysis illustrating the relationship between maize stalk lodging resistance characteristics (red arrows) and lodging properties (blue arrows). **(C)** Relationship between stalk lodging resistance index and lodging rate. **(D)** Relationship between maize stalk lodging resistance characteristics and lodging properties. LI, stalk lodging resistance index; PH, plant height; EH, ear height; GH, gravity height; EHC, ear height coefficient; GHC, gravity height coefficient; BS, bending strength; RPS, rind penetration strength; IL, internode length; ID, internode diameter; LDR, length-diameter ratio; MC, moisture content; IP, internode plumpness. Solid lines indicate a significant or highly significant linear relationship, and dashed lines indicate that the linear relationship is not significant. * and ** indicate that correlation is significant at the P < 0.05 and P < 0.01 levels, respectively, with no markings indicating a non-significant correlation. CK, control treatment; SV6, spraying EDAH at V6; SV12, spraying EDAH at V12; TS, twice-spraying EDAH; D_4.5_, planting density of 4.5 × 10^4^ plants ha^-1^; D_5.25_, planting density of 5.25 × 10^4^ plants ha^-1^; and D_8.25_, planting density of 8.25 × 10^4^ plants ha^-1^.

**Figure 3:**
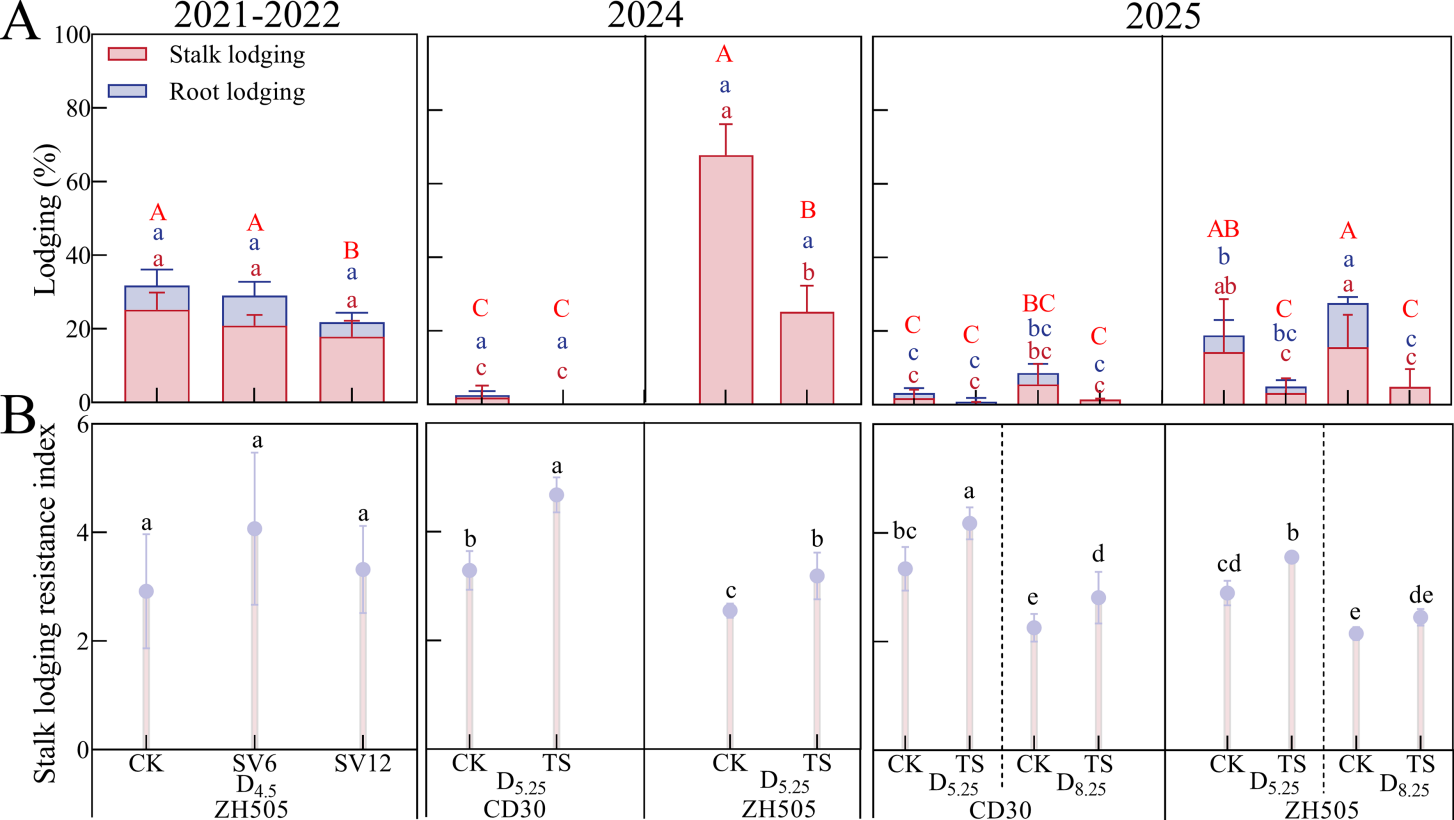
Lodging rate **(A)** and stalk lodging resistance index **(B)** in 2021–2022 and 2024–2025. Vertical bars display the standard errors. Different lowercase letters indicate significant differences (*P < 0.05*) in stalk lodging rate, root lodging rate, and stalk lodging resistance index between different treatments, as determined by the LSD multiple range test. Different uppercase letters in plot A indicate significant differences in lodging rate. CK, control treatment; SV6, spraying EDAH at V6; SV12, spraying EDAH at V12; TS, twice-spraying EDAH; D_4.5_, planting density of 4.5 × 10^4^ plants ha^-1^; D_5.25_, planting density of 5.25 × 10^4^ plants ha^-1^; D_8.25_, planting density of 8.25 × 10^4^ plants ha^-1^.

Single-spraying EDAH had no significant overall impact on grain yield or its components (**Figures 1**, **2A**; **Supplementary Table 2**). The effects of TS varied across different years, hybrids, and planting densities. In 2024, the grain yield of the lodging-resistant hybrid CD30 was not significantly increased by TS, whereas grain yield increased by 18.04% in the lodging-susceptible hybrid ZH505 (**Figure 1D**). This increase was likely attributed to enhanced stalk lodging resistance (**Figure 3B**), which mitigated severe lodging in ZH505 (**Figure 3A**) caused by strong winds (18 m s^-1^) on 1 July (**Supplementary Figure 1**). In 2025, no strong winds occurred during the filling stage, resulting in lower lodging rates and only a slight yield enhancement (2.79%) (**Figure 1D**). Regarding grain yield components, TS exhibited a certain promoting effect on 100-grain weight or grains per ear at a planting density of 5.25 × 10^4^ plants ha^−1^ (D_5.25_) (**Figure 1B**), and increased the number of effective ears at a planting density of 8.25 × 10^4^ plants ha^−1^ (D_8.25_) (**Figure 1C**). Grain yield increased by 4.94% with increasing planting density (**Figure 1D**). Correlation pathway analyses revealed that 100-grain weight contributed the most to grain yield (60.51%–89.08%) at D_4.5_ and D_5.25_, whereas the number of effective ears contributed the most (68.90%) at D_8.25_.

Spraying EDAH reduced the lodging rate, particularly with twice-spraying (**Figure 3**). In 2021–2022, single-spraying EDAH at V6 and V12 (SV6 and SV12) reduced the lodging rate by only 2.80–7.24 percentage points. The lodging rate was significantly reduced by 11.57–22.54 percentage points following twice-spraying EDAH in 2024–2025 (**Figures 3A**, **2A**; **Supplementary Table 2**). Twice-spraying EDAH, in combination with CD30, maintained lodging rates at 0.00-1.44% and the stalk lodging resistance index (LI) at 2.81–4.68. Twice-spraying EDAH increased grain yield while reducing lodging rates (**Figures 1**, **3**, **2A**).

### 3.2 Plant morphological traits, third internode mechanical strengths, and leaf angles

**Figure 4:**
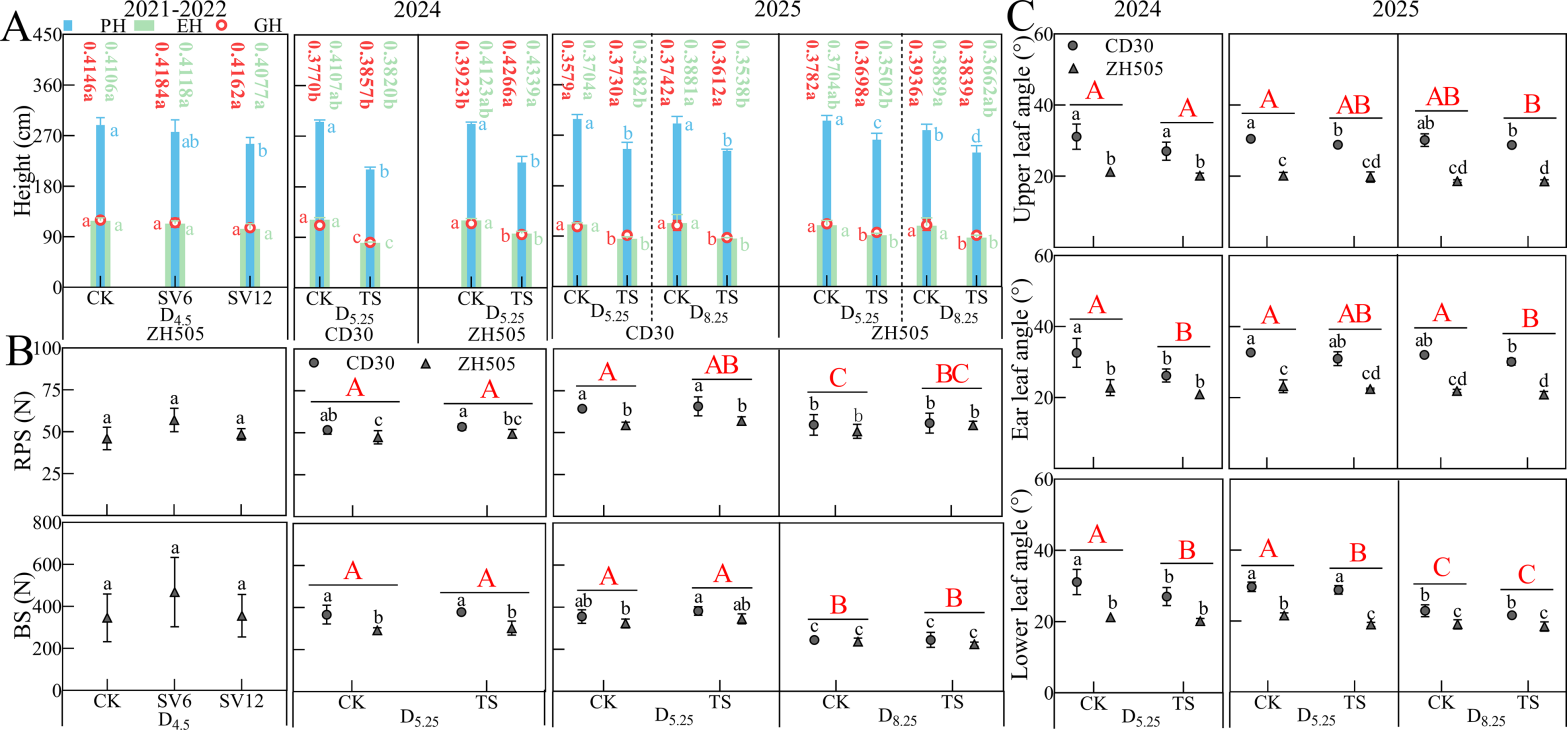
Plant morphology **(A)**, internode mechanical strength **(B)**, and leaf angle **(C)** in 2021–2022 and 2024–2025. Vertical bars display the standard errors. In plot A, green and red numbers above the columns indicate ear height coefficients and gravity height coefficients under the corresponding treatment, respectively. Different lowercase letters indicate significant differences (*P < 0.05*) between different treatments, and different uppercase letters in plots B and C indicate differences in plant growth retardant treatments, with the LSD multiple range test. PH, plant height; EH, ear height; GH, gravity height; BS, bending strength; RPS, rind penetration strength; CK, control treatment; SV6, spraying EDAH at V6; SV12, spraying EDAH at V12; TS, twice-spraying EDAH; D_4.5_, planting density of 4.5 × 10^4^ plants ha^-1^; D_5.25_, planting density of 5.25 × 10^4^ plants ha^-1^; D_8.25_, planting density of 8.25 × 10^4^ plants ha^-1^.

Single-spraying EDAH significantly optimized plant morphology and enhanced internode mechanical strength (**Figure 4**; **Supplementary Tables 2, 3**). Compared with CK, SV6 and SV12 reduced plant height (PH), ear height (EH), and gravity height (GH) by 4.37% and 11.60%, 4.41% and 12.03%, and 3.67% and 11.03%, respectively (**Figure 4A**). Regarding mechanical strength, increases under SV6 (23.93%–35.48%) were significantly greater than those under SV12 (2.82%–5.43%) (**Figure 4B**). PH, EH, and GH were reduced by 15.10%–26.20%, 20.79%–26.74%, and 16.04%–21.99%, respectively, under TS. The variations in ear height coefficient (EHC) and gravity height coefficient (GHC) were relatively small (**Figure 2A**). TS increased rind penetration strength (RPS) and bending strength (BS) by only 3.14%–3.53% (**Figures 4B**, **2A**; **Supplementary Table 3**). In contrast, planting density and hybrid effects were more pronounced. Mechanical strengths at D_8.25_ were significantly reduced compared to D_5.25_, and the reduction in BS (32.45%) was greater than that of RPS (10.68%). CD30 exhibited greater RPS and BS by 10.48% and 17.37% than ZH505, respectively. Increasing planting density and TS both significantly reduced leaf angles (**Figure 4C**; **Supplementary Table 2**). The leaf angle at the third leaf below-ear showed the greatest reduction, followed by the ear leaf, whereas the smallest reduction was observed at the third leaf above-ear. The hybrid effects were greater than those of planting density and TS (**Supplementary Table 2**). Averaging leaf angles, the leaf angle of CD30 was 38.27% higher than that of ZH505.

### 3.3 Internode morphological traits and matter constituents

**Figure 5:**
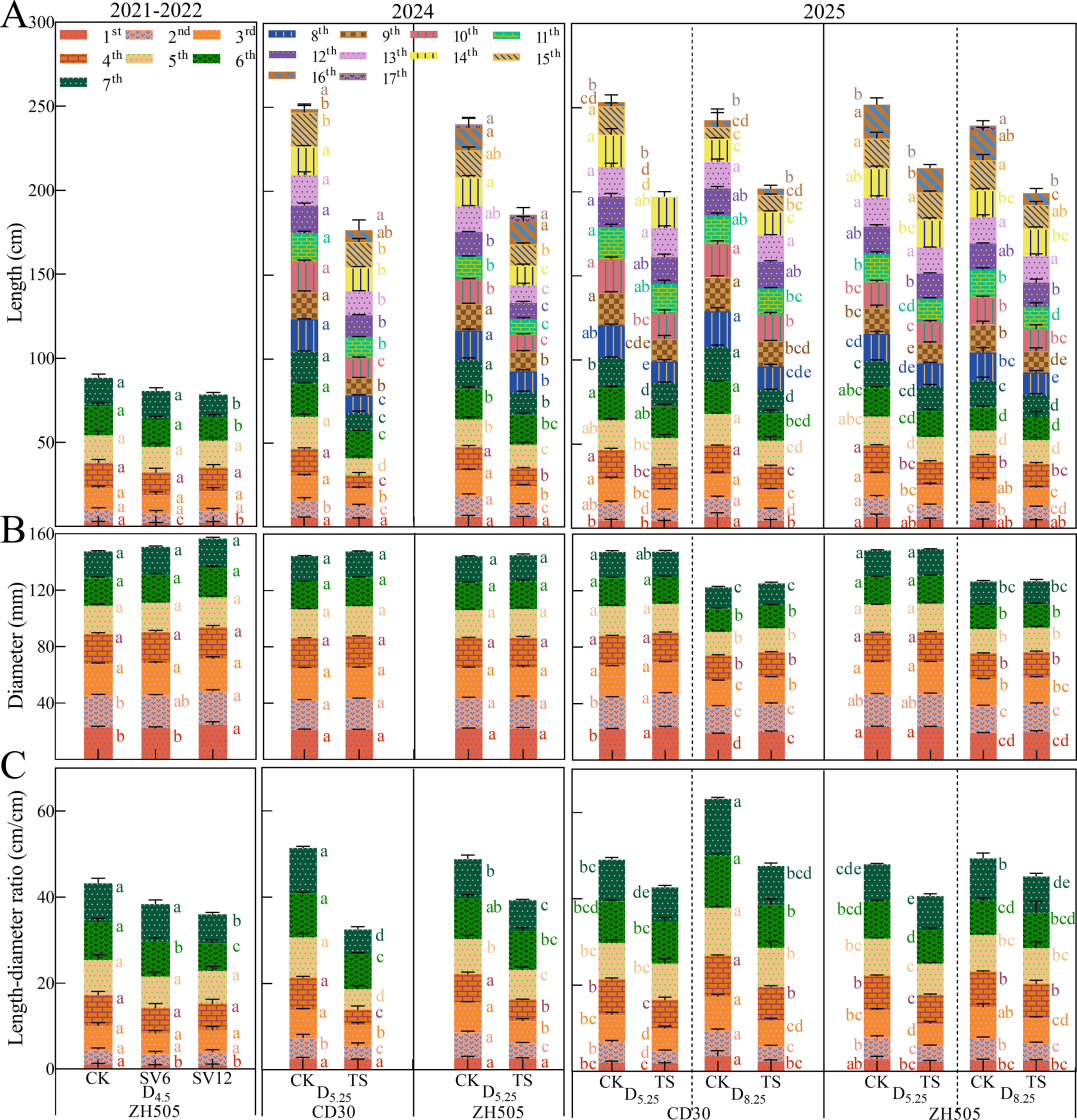
Internode morphological traits: **(A)** internode length; **(B)**, internode diameter; and **(C)** internode length–diameter ratio in 2021–2022 and 2024–2025. Vertical bars display the standard errors. Different lowercase letters indicate significant differences (P < 0.05) between different treatments, with the LSD multiple range test. CK, control treatment; SV6, spraying EDAH at V6; SV12, spraying EDAH at V12; TS, twice-spraying EDAH; D_4.5_, planting density of 4.5 × 10^4^ plants ha^-1^; D_5.25_, planting density of 5.25 × 10^4^ plants ha^-1^; and D_8.25_, planting density of 8.25 × 10^4^ plants ha^-1^.

**Figure 6:**
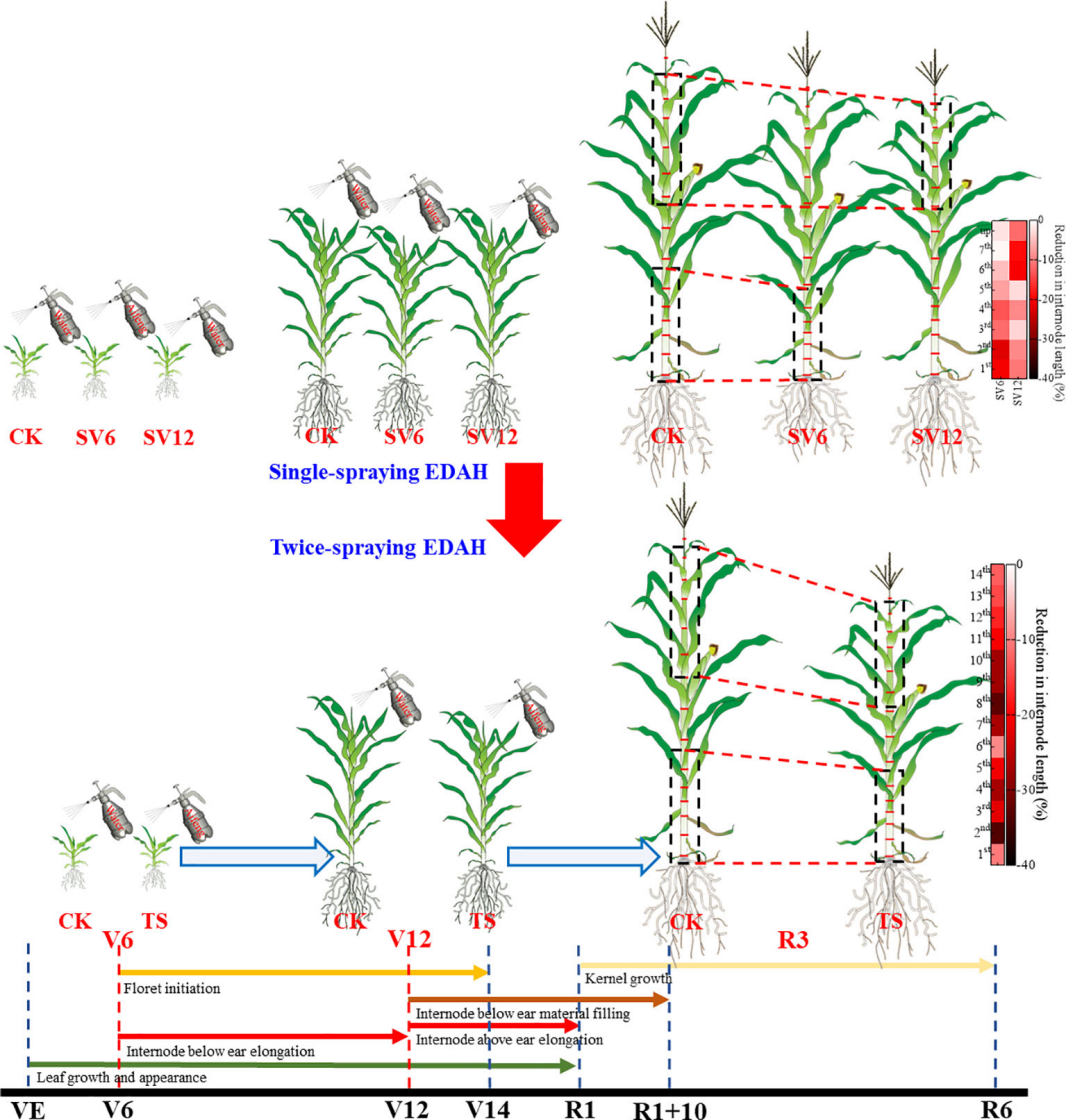
Schematic diagram of the effect of single- and twice-spraying the plant growth regulator EDAH. CK, control treatment; SV6, spraying EDAH at V6; SV12, spraying EDAH at V12; and TS, twice-spraying EDAH.

**Figure 7:**
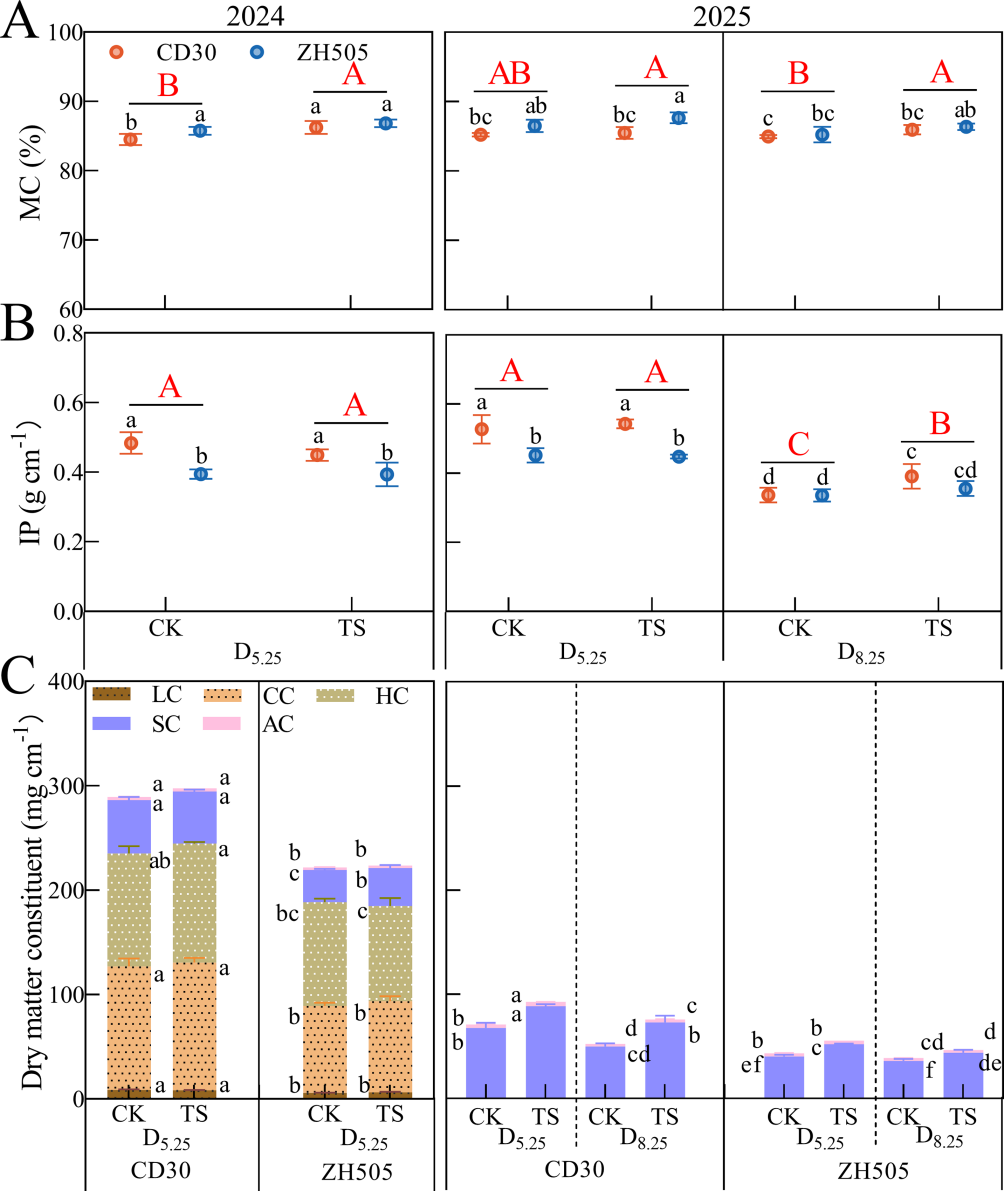
Internode moisture content **(A)**, internode plumpness **(B)**, and dry matter constituents **(C)** in 2024–2025. Vertical bars display the standard errors. MC, moisture content; IP, internode plumpness. LC, lignin content; CC, cellulose content; HC, hemicellulose content; SC, soluble sugar content; AC, amylum content. Different lowercase letters indicate significant differences (P < 0.05) between different treatments, and different uppercase letters in plots A and B indicate differences in plant growth retardant treatments, with the LSD multiple range test. CK, control treatment; TS, twice-spraying EDAH; D_5.25_, planting density of 5.25 × 10^4^ plants ha^-1^; and D_8.25_, planting density of 8.25 × 10^4^ plants ha^-1^.

Single- and twice-spraying EDAH, planting density, and hybrid all significantly influenced internode morphology (**Figure 5**; **Supplementary Table 3**). SV6 significantly reduced the first to the fourth internode length (IL), while SV12 mainly reduced the IL of the sixth and higher internodes (**Figures 4A**, **5A**, **6**). TS significantly reduced IL (17.66%–25.79%), particularly in the second to the fifth and the seventh to the eleventh internodes. In contrast, internode diameter (ID) increased only slightly (0.69%–1.40%), resulting in a significant reduction in the length–diameter ratio (LDR, 16.14%–28.36%) (**Figures 5A**, **2A**, **6**; **Supplementary Table 3**). As planting density increased from D_5.25_ to D_8.25_, IL and ID decreased by 3.59% and 15.52%, respectively, leading to a 13.68% increase in LDR. Under TS, moisture content (MC) significantly increased by 1.03%–1.64%. Internode plumpness (IP) did not show a significant increase at D_5.25_ but increased by 5.22% at D_8.25_ (**Figures 7A, B**; **Supplementary Table 3**). Compared with ZH505, IP in CD30 was 15.66% higher, particularly at D_5.25_. MC of CD30 was also 1.13% lower than that of ZH505. Increasing planting density had no significant effect on MC but significantly reduced IP. Regarding internode dry matter constituents, the effects were significantly greater than those of TS (**Figure 7C**; **Supplementary Table 3**). CD30 exhibited higher lignin content (LC), cellulose content (CC), hemicellulose content (HC), soluble sugar content (SC), and amylum content (AC) than ZH505 (**Figure 7C**; **Supplementary Table 3**). Among the measured dry matter constituents, SC showed the greatest increase under TS (5.09%–32.86%).

### 3.4 Correlation analysis

**Figure 8:**
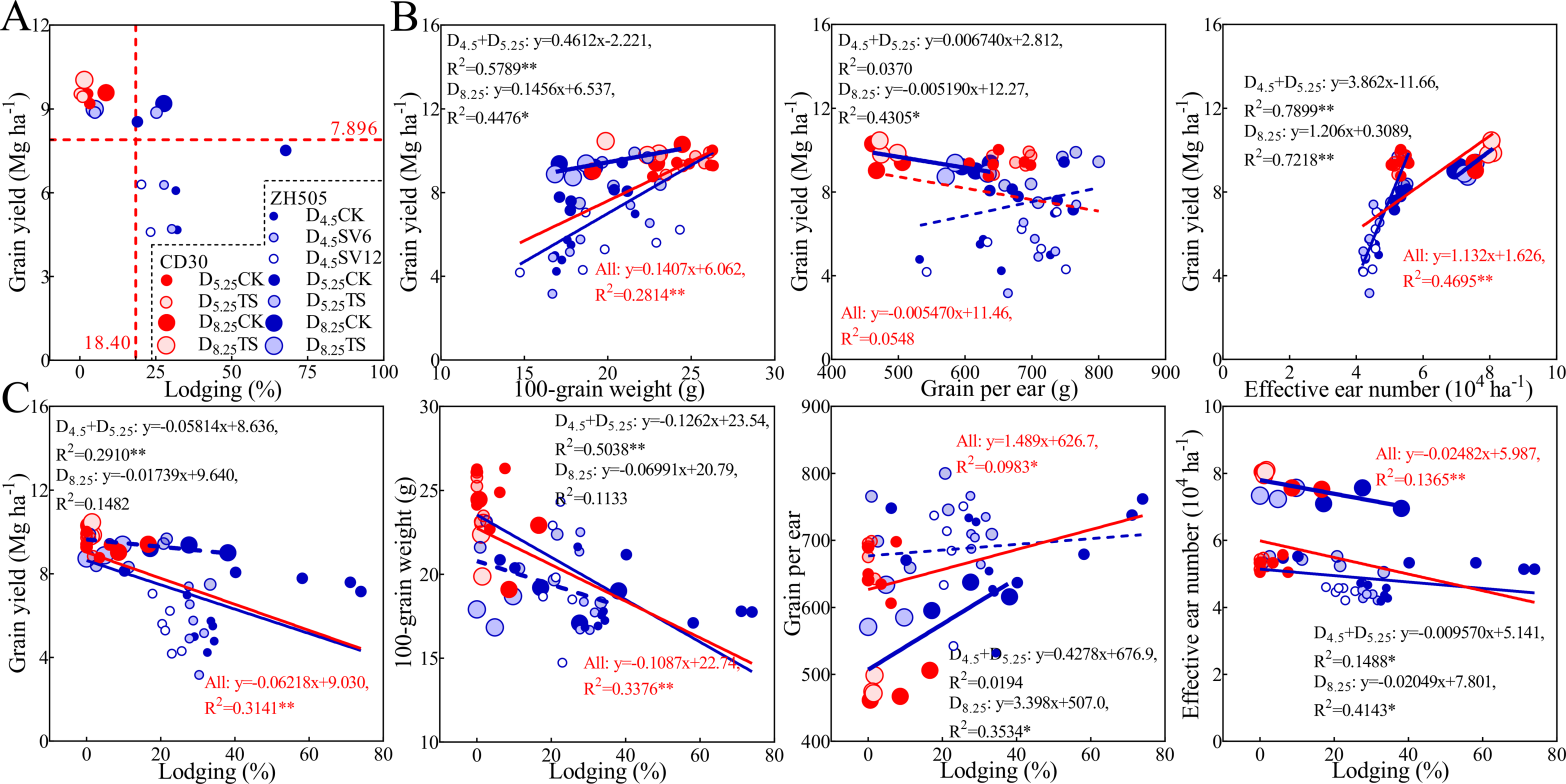
Distribution of lodging and grain yield **(A)**, the linear relationship between grain yield components and grain yield **(B)**, and the linear relationship between lodging, grain yield, and its components **(C)**. The red lines indicate the linear relationship at 4.50–8.25 × 10^4^ plants ha^-1^ planting density in 2021–2022 and 2024–2025; the thinner blue lines indicate the linear relationship at 4.50 and 5.25 × 10^4^ plants ha^-1^ planting density; and the thicker blue lines indicate the linear relationship at 8.25 × 10^4^ plants ha^-1^ planting density. Solid lines indicate a significant or highly significant linear relationship, while dashed lines indicate that the linear relationship is not significant. * and ** indicate that correlation is significant at the P < 0.05 and P < 0.01 levels, respectively, and no markings indicate that correlation is not significant. CK, control treatment; SV6, spraying EDAH at V6; SV12, spraying EDAH at V12; TS, twice-spraying EDAH; D_4.5_, planting density of 4.5 × 10^4^ plants ha^-1^; D_5.25_, planting density of 5.25 × 10^4^ plants ha^-1^; and D_8.25_, planting density of 8.25 × 10^4^ plants ha^-1^.

Spraying EDAH and the lodging-resistant hybrid (CD30) resulted in higher grain yield and greater grain yield stability, along with lower lodging rates (**Figure 8A**). Generally, lodging rate negatively affected grain yield and its components (**Figure 8C**). Lodging rate was negatively correlated with LI (**Figures 2B, C**). LI was significantly positively correlated with BS and RPS, but negatively correlated with plant morphologies (**Figures 2B, D**). Lodging rate showed significant or highly significant positive correlations with PH, EH, and GH, while exhibiting a negative but non-significant correlation with BS and RPS. Redundancy analysis indicated that stalk lodging resistant traits explained 62.87% of the variation in lodging properties, and plant morphologies explained 50.10%, with GH contributing 40.00% (**Figure 2B**). GH was jointly influenced by PH and EH (**Supplementary Figure 3**). Stepwise regression analysis of PH (X_PH_) and EH (X_EH_) with GH (Y_GH_) yielded the equations: Y_GH_ = 26.39 + 0.7792X_EH_ (R^2^, 0.8974**) and Y_GH_ = 16.83 + 0.1264X_PH_ + 0.4989X_EH_ (R^2^, 0.9548**) for 2021–2022 and 2024–2025, respectively. In 2021–2022, reductions in PH and GH were closely associated with IL of the sixth and seventh internodes. ILs of the second to the fifth and the seventh to the twelfth internodes were more strongly associated with PH than other internodes, while ILs of the second to the fifth and the seventh internodes were more strongly associated with EH in 2024–2025 (**Figure 2A**; **Supplementary Figure 4**).

## 4 Discussion

### 4.1 Twice-spraying EDAH synergistically improved lodging resistance and grain yield

Effects of plant growth regulators on maize stalk lodging resistance and grain yield depended not only on regulator type (Xu et al., 2017; Reis et al., 2020), but also on spraying stages (Ren et al., 2024), concentration (Li et al., 2020), and timing (Zhao et al., 2022a; Ren et al., 2024). Single-spraying EDAH at V6 or V12 improved stalk lodging resistance by optimizing plant morphology and enhancing internode mechanical strength (**Figure 4**), but it was insufficient to effectively reduce lodging (**Figure 3**). Twice-spraying EDAH (first at V6 and second at V12) significantly reduced PH, EH, and leaf angle (**Figures 4A, C**; **Supplementary Figure 5**), thereby decreasing wind resistance (Xue et al., 2020). Twice-spraying EDAH also reduced leaf and stalk dry matter (**Supplementary Figure 2**), thereby lessening gravitational stress and bending moment acting on basal internodes (Robertson et al., 2017; Xue et al., 2020), and consequently significantly reducing stalk lodging rate (**Figure 3A**). In this study, plant morphology explained 60.10% of the variation in lodging properties, with GH as a key factor (40.00%) (**Figures 2B, D**) jointly determined by EH and PH (**Supplementary Figure 3**). However, twice-spraying EDAH slightly increased internode mechanical strength by 3.34% (**Figures 4B**, **7**, **2A**), which differed from previous studies (Xu et al., 2017; Huang et al., 2021). ETH could stimulate or inhibit plant growth depending on doses, developmental stages, and organs (Reis et al., 2020; Zhang et al., 2020). In strawberry, ETH promoted material accumulation in early-development stage fruits (green fruits), while inhibiting it in late-development stage fruits (pink and red fruits) (Reis et al., 2020). ETH promoted cell wall degradation in “Nanguo” pear fruits (Jiang et al., 2024) and downregulated genes associated with secondary cell wall formation in rubber trees (Nakano et al., 2021). The growth stage from V12 to silking was critical for basal internode material filling, which was essential for developing mechanical strength (Zhan et al., 2022), and material filling might be limited by spraying ETH again at V12. Therefore, enhancing stalk lodging resistance under TS primarily resulted from optimizing plant morphology (particularly GH), rather than enhancing internode filling capacity. Additionally, timely spraying EDAH suppressed aboveground growth while promoting root growth, increasing the diameter, volume, dry weight, and number of roots (Ali et al., 2024). This study observed that TS enhanced the number of brace root whorls (**Supplementary Figure 6**), thereby reducing root lodging rate (**Figure 3A**).

Maize grain yield response to plant growth regulators also depends on whether lodging occurs (Zhao et al., 2022b). At V7, spraying N, N-Diethyl-2-hexanoyl oxygen radicals-ethyl amine phosphonic acid salt (DHEAP) improved the middle-canopy light environment by increasing the upper-canopy leaf tilt angle, thereby increasing grain yield (22.28%–43.14%) at high density, but not at low density (Huang et al., 2021). Additionally, spraying EDAH increased grain yield by 14.30% by reducing the upper-middle canopy leaf area to optimize the lower-middle canopy light environment (Xu et al., 2017). At V14, spraying a high-concentration mixture (ETH and cycocel, w/w = 3:1; EC) increased grain yield (8.10%) by shortening the distance between upper leaves and the ear (Ren et al., 2024). In this study, the reduction in lodging rate was limited after a single application of EDAH, with grain yield increasing by only 1.29%–2.16%. However, twice-spraying EDAH significantly reduced the lodging rate while increasing grain yield by 5.01%, particularly in lodging-susceptible hybrids under conditions conducive to lodging, such as strong winds with heavy rain (**Figures 1D**, **3A**, **8**, **2A**). The reduction in lodging rate increased the effective ear number (Li et al., 2025), especially at D_8.25_ (**Figures 1C**, **8**), and prevented significant declines in 100-grain weight and grains per ear at D_5.25_ (Zhang et al., 2017; Li et al., 2025; **Figures 1A, B**, **8**). A more compact plant structure (**Figure 4C**) not only reduced wind resistance but also enhanced ventilation and promoted photosynthetically active radiation interception in lower-middle canopy leaves (Huang et al., 2021, 2024), particularly at higher densities. This facilitated dry matter accumulation (Xu et al., 2017). Although the growth and development of leaves and stalks were inhibited by twice-spraying EDAH (**Supplementary Figure 2**), this could be attributed to ETH (Zhang et al., 2019; Geng et al., 2022). However, the combination of ETH with DA-6 enhanced the leaves’ photosynthetic capacity, as DA-6 increased chlorophyll, protein, and nucleic acid content in leaves (Xu et al., 2017; Hong et al., 2025). Furthermore, DA-6 also increased root activity (Ali et al., 2024). Although total plant dry matter accumulation decreased by 9.71%–13.86%, assimilate allocation to the ear increased (8.24%–9.42% higher than CK) (**Supplementary Figure 2**; Zhao et al., 2022a).

### 4.2 Maize stalk formation was stage-regulated by twice-spraying EDAH

Maize has taller stalks with more internodes than wheat and rice, necessitating distinct architectural regulation strategies. Rapid elongation of the first to seventh basal internodes occurs during V6–V12 (approximately 20 days) (Zhan et al., 2022). After V12, rapid elongation shifts to the upper internodes (approximately 10 days) (Ren et al., 2024). Thus, maize internode elongation spans approximately 30 days. However, the regulatory duration of a single application of plant growth regulators is insufficient to cover this entire elongation period; for example, the effect of EC lasted only 15 days (Tang et al., 2022). Applying plant growth regulator during the initial elongation stage of maize basal internodes (V6–V8) significantly inhibited only basal internode elongation (Xu et al., 2017; Huang et al., 2021; **Figures 5A**, **2A**). However, no reshaping effect on the internodes above the ear was observed at concentrations that did not inhibit grain yield (Li et al., 2020; Ren et al., 2024). In some cases, a compensatory “rebound effect” was observed, characterized by an increase in the length of the internodes above the ear (Huang et al., 2024). In contrast, single-spraying EDAH at V12 shortened only the internode lengths at the middle-upper sections (**Figures 5A**, **2A**, **6**). Hong et al. (2025) and Ren et al. (2024) applied EDAH and EC at V14, which shortened internode lengths above the ear. By contrast, twice-spraying EDAH coordinately reduced the elongation of both basal internodes and upper internodes above the ear (**Figures 5A**, **2A**, **6**; **Supplementary Table 3**). Consequently, twice-spraying EDAH achieved greater reductions in PH, EH, and GH (20.67%, 23.90%, and 19.06%, respectively) (**Figures 4A**, **2A**; **Supplementary Figure 5**). Previous studies showed that single-spraying EDAH, DHEAP, or EC at different developmental stages mainly reduced either basal or upper internode elongation, resulting in limited reshaping of overall plant architecture (Xu et al., 2017; Huang et al., 2021; Hong et al., 2025; Ren et al., 2024; **Figure 4A**). This indicated that single-spraying plant growth regulator, whether during the early or mid-late stages of internode elongation, was less effective for reshaping plant morphologies than twice-spraying. Therefore, twice-spraying EDAH provides a stage-specific regulation strategy and theoretical support for coordinated maize dwarfing and architectural optimization.

### 4.3 Potential optimization strategy for twice-spraying EDAH

The grain yield advantage of twice-spraying EDAH was primarily attributed to reduced lodging rather than to increases in grain per ear or 100-grain weight (**Figure 8**). To further increase grain yield, it is necessary to unlock the potential of grain per ear and grain weight, as shown in previous studies (Wang et al., 2024; Xu et al., 2023). Maize ear development and internode elongation exhibit a coordinated growth relationship (Xu et al., 2004); therefore, spraying ETH restricts both internode elongation (Liu et al., 2025) and grain yield formation (Zhao et al., 2022b). Minimizing the negative effect of ETH on reproductive organs was key to synergistically improving stalk lodging resistance and grain yield (Wang et al., 2025). Maize floret initiation, which determines grain per ear, lasts from approximately V6–V14 (Zhao et al., 2022b; Ren et al., 2024). Although ETH inhibits flower primordia formation, DA-6 could compensate for this negative effect, particularly with dense planting (Wang et al., 2025). In this study, ETH and DA-6 were active ingredients in this plant growth regulator. Twice-spraying stages were conducted during the floret initiation stage (V6 and V12), without reducing grain per ear (**Figure 1B**). The shortening of internodes in or above the ear prevented competition between stalk and ear development, thereby promoting assimilate allocation to the ear (Ren et al., 2024; Zhao et al., 2022a). Our results were consistent with this finding (**Supplementary Figure 2**). Delaying the second spraying until V14 to avoid the negative effect of ETH on floret initiation and suppressed internode elongation above the ear (Ren et al., 2024) might unlock the potential for increased grain per ear. Spraying DA-6 at VT, after spraying EDAH at V7, could overcome the negative effect (Gong et al., 2021). Delaying the second spraying time to V14 and increasing DA-6 concentration may further enhance the synergistic regulation of maize grain yield and stalk lodging resistance.

## 5 Conclusions

This study proposed and established the EDAH spraying technical model in a maize planting system, involving twice-spraying EDAH (first at V6 and second at V12) to regulate maize stalk growth in stages, which significantly reduced lodging rate (64%–79%). Twice-spraying EDAH synchronously shortened internode length below and above the ear, vastly lowering PH, EH, and GH by 19.06%–23.90%. However, there was no significant improvement in the basal internode mechanical strengths, diameter, or dry matter constituents. This strategy achieved synergistic improvement in stalk lodging resistance and grain yield without harming ear development. This research provided new insight and theoretical support for formulating plant growth regulator strategies in intensified maize production.

## Data Availability

The original contributions presented in the study are included in the article/**Supplementary Material**. Further inquiries can be directed to the corresponding authors.
