# Supplementary material for: Twice-spraying plant growth regulator EDAH stage-regulates maize plant morphology: a novel strategy for enhancing stalk lodging resistance
Source: Front Plant Sci. 2026 Jun 23;17:1853755. doi: 10.3389/fpls.2026.1853755 (PMC13337377; doi:10.3389/fpls.2026.1853755)
Supplement: Supplementary file 1 [file Supplementaryfile1.pdf]

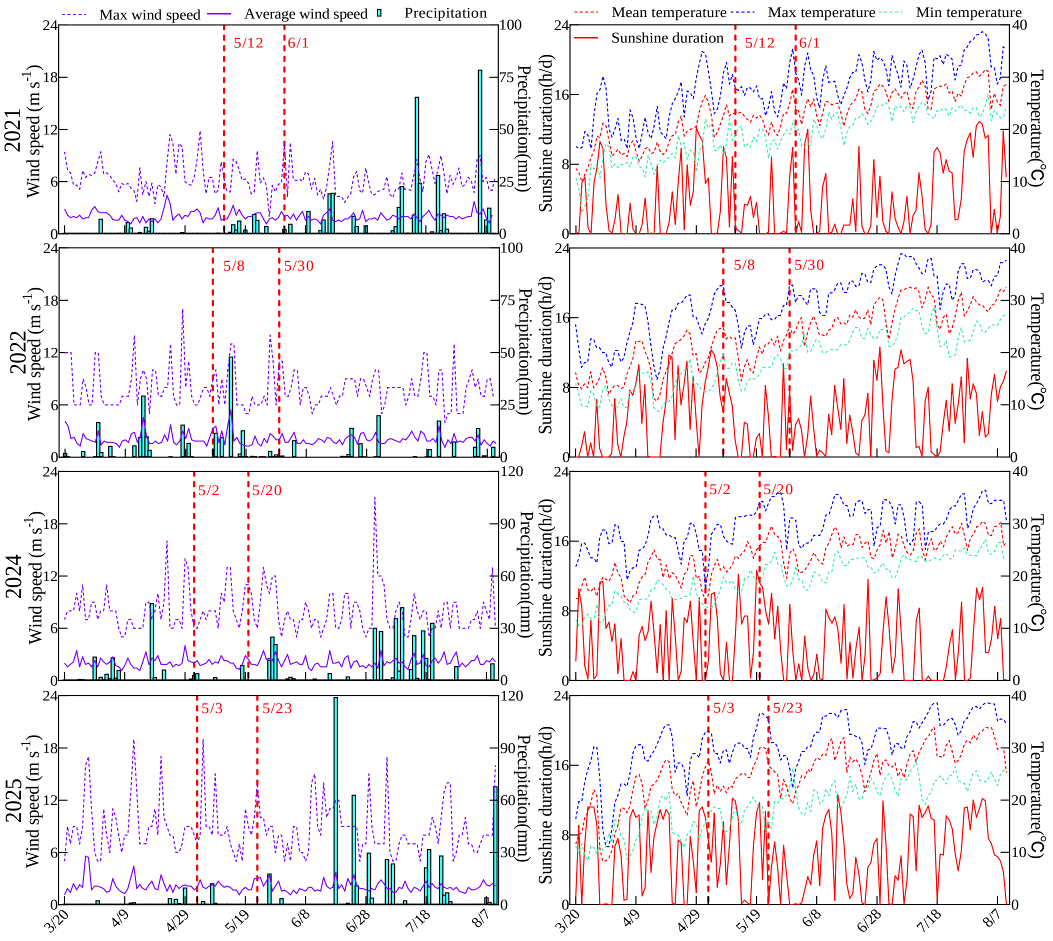

**Fig. S1** Daily temperature, sunshine duration, precipitation and wind speed during the 2021-2022 and 2024-2025 growing seasons. The red dotted line marks the application time of the plant growth retardant EDAH.

**Table S1**

Sowing schedules and reproductive processes in 2021-2022 and 2024-2025.

| Year | Sowing | Emergence | V6 | V12 | Silking | Maturity |
|---|---|---|---|---|---|---|
| 2021 | 3/30 | 4/10 | 5/12 | 6/1 | 6/14 | 8/4 |
| 2022 | 3/25 | 4/8 | 5/8 | 5/30 | 6/9 | 7/31 |
| 2024 | 3/27 | 4/4 | 5/2 | 5/20 | 5/27 | 7/15 |
| 2025 | 3/28 | 4/6 | 5/3 | 5/23 | 6/2 | 7/19 |

**Table S2**

Results of an analysis of variance test on repeated measurements of the grain yield, lodging, plant morphology, leaf angle and dry matter accumulation in 2021-2022 and 2024-2025.

| Indexes | | 2021-2022 F test (Y) | F test (PGR) | F test (Y×PGR) | 2024 F test (PGR) | F test (H) | F test (PGR×H) | 2025 F test (D) | F test (PGR) | F test (H) | F test (D×PGR) | F test (D×H) | F test (PGR×H) | F test (D×PGR×H) |
|---|---|---|---|---|---|---|---|---|---|---|---|---|---|---|
| Grain yield (Mg ha$^{-1}$) | | 37.23** | 0.07 | 0.11 | 4.62 | 18.14** | 4.60 | 12.08** | 2.61 | 26.61** | 0.34 | 0.16 | 1.25 | 2.08 |
| 100-grain weight (g) | | 7.09* | 0.89 | 0.38 | 2.15 | 135.40** | 7.77* | 29.91** | 0.00 | 49.09** | 1.12 | 1.53 | 0.10 | 0.33 |
| Grain per ear | | 0.40 | 1.30 | 0.00 | 1.19 | 8.06* | 0.00 | 93.88** | 0.19 | 39.95** | 1.10 | 9.15** | 0.11 | 0.27 |
| Effective ear number ($10^4$ ha$^{-1}$) | | 6.34* | 0.47 | 0.08 | 0.58 | 0.58 | 0.00 | 726.35** | 1.91 | 11.27** | 2.82 | 14.82** | 0.16 | 0.24 |
| Lodging (%) | | 2.32 | 20.06** | 0.36 | 41.18** | 165.94** | 32.50** | 1.80 | 18.17** | 15.05** | 1.58 | 0.062 | 6.28* | 0.13 |
| Stalk lodging resistance index | | 252.12** | 39.38** | 8.90** | 49.40** | 59.60** | 6.79* | 105.62** | 31.68** | 13.35** | 2.45 | 2.10 | 1.04 | 0.034 |
| Plant height (cm) | | 49.34** | 30.20** | 1.85 | 425.92** | 1.25 | 4.67 | 13.29** | 155.38** | 0.01 | 0.02 | 3.97 | 4.16 | 0.60 |
| Ear height (cm) | | 6.94* | 9.31** | 1.65 | 315.88** | 17.25** | 22.69** | 0.06 | 86.01** | 0.07 | 0.31 | 0.61 | 1.93 | 0.01 |
| Gravity height (cm) | | 0.34 | 3.38 | 0.54 | 380.10** | 43.38** | 17.90** | 1.77 | 100.06** | 4.91* | 1.85 | 0.53 | 0.29 | 0.14 |
| Ear height coefficient (%) | | 0.57 | 0.03 | 1.92 | 0.31 | 17.27** | 15.19** | 4.27 | 12.60** | 0.29 | 0.27 | 0.16 | 0.23 | 0.12 |
| Gravity height coefficient (%) | | 2.87 | 0.02 | 0.93 | 14.06** | 23.94** | 4.95 | 2.26 | 0.51 | 6.87* | 1.69 | 1.23 | 0.80 | 1.41 |
| Upper leaf angle (°) | | | | | 3.57 | 37.91** | 1.22 | 4.50 | 5.30* | 718.86** | 0.13 | 2.50 | 2.78 | 0.00 |
| Ear leaf angle (°) | | | | | 6.03* | 20.02** | 1.81 | 5.82* | 8.98** | 419.01** | 0.05 | 0.53 | 1.05 | 0.00 |
| Lower leaf angle (°) | | | | | 8.98* | 13.94** | 0.28 | 78.07** | 7.74* | 169.25** | 0.70 | 31.75** | 0.31 | 1.57 |
| Dry matter accumulation (g plant$^{-1}$) | Leaf | 27.68** | 3.50 | 3.01 | 7.62* | 0.38 | 4.41 | 288.08** | 77.22** | 8.51* | 3.19 | 0.60 | 0.54 | 1.86 |
| | Stalk+sheath | 76.99** | 2.48 | 0.74 | 35.79** | 6.25* | 6.61* | 130.69** | 31.95** | 0.00 | 1.50 | 3.47 | 0.16 | 3.28 |
| | Ear | 60.16** | 5.48* | 1.86 | 3.67 | 0.02 | 1.24 | 50.65** | 0.28 | 0.0044 | 0.17 | 0.00 | 1.45 | 0.42 |
| | Tassel+bract | 25.47** | 2.12 | 0.48 | 2.95 | 14.85** | 0.09 | 164.60** | 0.065 | 47.36** | 2.17 | 0.00 | 0.44 | 1.26 |

Note: * and ** represent significant differences at $P < 0.05$ and $P < 0.01$, respectively. Y, year; PGR, spraying plant growth regulator EDAH; H, hybrid; D, density.

**Table S3**

Results of an analysis of variance test on repeated measurements of the internode morphology, internode mechanical strength and matter constituent in 2021-2022 and 2024-2025.

| Indexes | | 2021-2022 | | | 2024 | | | 2025 | | | | | | |
|---|---|---|---|---|---|---|---|---|---|---|---|---|---|---|
| | | F test (Y) | F test (PGR) | F test (Y×PGR) | F test (PGR) | F test (H) | F test (PGR×H) | F test (D) | F test (PGR) | F test (H) | F test (D×PGR) | F test (D×H) | F test (PGR×H) | F test (D×PGR×H) |
| Length (cm) | 1st internode | 0.98 | 1.48 | 0.00 | 0.35 | 4.02 | 0.00 | 0.03 | 4.42 | 0.92 | 0.03 | 7.02* | 0.39 | 2.67 |
| | 2nd internode | 0.37 | 2.31 | 0.95 | 60.75** | 7.94* | 2.68 | 1.28 | 130.01** | 4.15 | 0.22 | 6.45* | 5.97* | 2.24 |
| | 3rd internode | 19.91** | 5.69* | 3.55 | 58.23** | 0.38 | 0.00 | 0.33 | 31.43** | 0.21 | 0.08 | 0.90 | 0.10 | 0.20 |
| | 4th internode | 6.85* | 10.49** | 11.19** | 65.80** | 0.24 | 11.34* | 6.61* | 95.35** | 3.90 | 4.92* | 0.10 | 7.43* | 2.56 |
| | 5th internode | 0.00 | 0.39 | 0.36 | 279.26** | 2.75 | 99.48** | 4.51 | 18.17** | 24.57** | 0.36 | 1.89 | 0.62 | 7.36* |
| | 6th internode | 13.86** | 41.37** | 1.89 | 21.92** | 0.04 | 11.76* | 9.23** | 4.71* | 29.56** | 0.22 | 1.19 | 0.64 | 2.84 |
| | 7th internode | 0.68 | 5.80* | 0.26 | 78.74** | 0.12 | 11.70* | 0.74 | 81.49** | 13.77** | 5.45* | 4.38 | 17.75** | 0.42 |
| | 8th internode | | | | 202.00** | 0.37 | 12.79* | 1.81 | 62.39** | 9.75** | 0.40 | 1.65 | 9.06** | 0.00 |
| | 9th internode | | | | 179.06** | 1.18 | 3.55 | 3.43 | 64.12** | 24.70** | 0.0030 | 0.09 | 2.32 | 0.22 |
| | 10th internode | | | | 109.22** | 24.48** | 4.10 | 0.83 | 28.77** | 23.03** | 0.16 | 0.06 | 1.80 | 0.32 |
| | 11th internode | | | | 84.17** | 45.82** | 0.00 | 6.21* | 15.45** | 16.14** | 0.10 | 2.91 | 2.81 | 0.10 |
| | 12th internode | | | | 57.43** | 21.18** | 0.87 | 1.69 | 3.55 | 2.98 | 0.74 | 0.01 | 0.01 | 0.27 |
| | 13th internode | | | | 26.52** | 14.65** | 0.54 | 5.04* | 0.68 | 0.99 | 0.42 | 0.91 | 0.20 | 0.75 |
| | 14th internode | | | | 100.52** | 11.23* | 12.36* | 19.38** | 0.18 | 0.04 | 1.10 | 11.53** | 0.040 | 0.00 |
| | 15th internode | | | | 13.39* | 6.90* | 0.66 | 0.13 | 10.53** | 30.45** | 8.93** | 0.18 | 2.28 | 14.90** |
| | 16th internode | | | | 0.68 | 8.18* | 0.66 | 0.82 | 14.26** | 93.22** | 0.25 | 8.34* | 7.14* | 1.86 |
| | 17th internode | | | | 0.48 | 4.84 | 0.48 | 3.34 | 3.34 | 3.34 | 3.34 | 3.34 | 3.34 | 3.34 |
| Diameter (mm) | 1st internode | 5.32 | 3.66 | 0.16 | 0.10 | 5.44 | 0.26 | 455.44** | 10.54** | 6.20* | 0.14 | 3.96 | 7.15* | 0.41 |
| | 2nd internode | 0.16 | 1.71 | 0.10 | 0.26 | 0.01 | 0.16 | 306.85** | 7.27* | 0.14 | 0.02 | 0.64 | 0.75 | 1.44 |

| | | | | | | | | | | | | | | |
|---|---|---|---|---|---|---|---|---|---|---|---|---|---|---|
| | 3rd internode | 0.00 | 1.30 | 0.70 | 0.01 | 3.18 | 0.06 | 496.56** | 2.55 | 7.08* | 0.20 | 0.18 | 2.27 | 1.05 |
| | 4th internode | 0.43 | 0.68 | 0.28 | 0.96 | 5.48 | 0.83 | 246.98** | 0.01 | 0.28 | 0.01 | 2.19 | 0.03 | 0.35 |
| | 5th internode | 2.10 | 2.84 | 1.63 | 1.45 | 2.27 | 0.24 | 151.99** | 0.00 | 0.32 | 0.06 | 2.84 | 0.89 | 0.88 |
| | 6th internode | 7.02* | 21.09** | 5.43* | 0.78 | 0.48 | 0.33 | 120.66** | 1.58 | 3.42 | 0.08 | 1.41 | 0.03 | 0.93 |
| | 7th internode | 0.84 | 23.23** | 4.27 | 0.84 | 1.11 | 0.28 | 45.64** | 0.09 | 3.54 | 0.17 | 0.35 | 0.24 | 1.36 |
| Length-diameter ratio | 1st internode | 0.65 | 1.88 | 0.04 | 0.53 | 1.48 | 0.020 | 4.07 | 5.01* | 0.21 | 0.19 | 5.29* | 0.94 | 2.55 |
| (cm/cm) | 2nd internode | 0.20 | 2.40 | 0.78 | 56.09** | 6.99* | 1.98 | 22.73** | 161.44** | 2.93 | 0.51 | 4.54 | 8.47* | 1.96 |
| | 3rd internode | 16.89** | 7.24* | 2.20 | 57.04** | 2.24 | 0.020 | 17.26** | 35.17** | 1.33 | 0.85 | 1.00 | 0.55 | 0.56 |
| | 4th internode | 2.00 | 8.77** | 4.06 | 72.14** | 1.15 | 12.42* | 41.21** | 69.47** | 4.50 | 1.57 | 1.58 | 7.03* | 4.18 |
| | 5th internode | 0.16 | 0.96 | 0.26 | 97.93** | 2.68 | 31.98** | 12.17** | 9.42** | 12.43** | 0.56 | 3.61 | 0.21 | 5.87* |
| | 6th internode | 23.42** | 63.66** | 0.05 | 14.37** | 0.10 | 7.46* | 1.63 | 1.71 | 23.97** | 0.35 | 2.57 | 0.54 | 3.02 |
| | 7th internode | 1.06 | 9.67** | 0.55 | 82.88** | 0.020 | 13.22* | 38.48** | 66.84** | 25.42** | 9.15** | 8.17* | 14.43** | 3.42 |
| Bending strength (N) | 3rd internode | 57.96** | 8.69** | 1.58 | 0.61 | 26.95** | 0.00 | 138.18** | 0.88 | 6.46* | 2.38 | 1.12 | 0.17 | 0.04 |
| Rind penetration strength (N) | 3rd internode | 11.64** | 29.28** | 4.49* | 0.67 | 14.73** | 0.37 | 15.59** | 1.80 | 13.24** | 0.01 | 4.33 | 0.34 | 0.04 |
| MC (%) | 3rd internode | | | | 13.19* | 5.61 | 0.72 | 3.71 | 8.05* | 11.16** | 0.32 | 4.83* | 0.71 | 0.37 |
| IPw ($g\ cm^{-1}$) | 3rd internode | | | | 1.75 | 30.24** | 1.64 | 186.48** | 4.56 | 26.13** | 2.39 | 11.09** | 1.82 | 0.14 |
| IPf ($g\ cm^{-1}$) | 3rd internode | | | | 4.45 | 13.77** | 0.16 | 156.03** | 14.22** | 1.60 | 1.31 | 0.30 | 0.10 | 0.86 |
| LC ($mg\ cm^{-1}$) | 3rd internode | | | | 0.07 | 103.99** | 3.39 | | | | | | | |
| CC ($mg\ cm^{-1}$) | 3rd internode | | | | 2.01 | 151.44** | 0.0044 | | | | | | | |
| HC ($mg\ cm^{-1}$) | 3rd internode | | | | 0.14 | 26.51** | 4.82 | | | | | | | |
| SC ($mg\ cm^{-1}$) | 3rd internode | | | | 2.47 | 157.72** | 6.10* | 59.27** | 117.82** | 330.17** | 0.09 | 12.72** | 17.06** | 1.09 |
| AC ($mg\ cm^{-1}$) | 3rd internode | | | | 5.28 | 60.26** | 0.022 | 201.53** | 20.12** | 14.04** | 3.25 | 7.37* | 17.29** | 0.54 |

Note: * and ** represent significant differences at $P < 0.05$ and $P < 0.01$, respectively. Y, year; PGR, spraying plant growth regulator EDAH; H, hybrid. D, density; MC, moisture content; IP, internode plumpness; LC, lignin content; CC, cellulose content; HC, hemicellulose; SC, soluble sugar content; AC, amylum content.

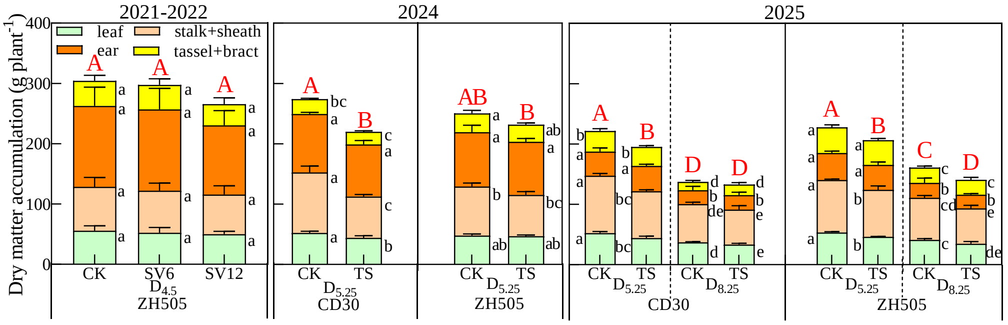


**Fig. S2** Dry matter accumulation per plant in 2021-2022 and 2024-2025. Different lowercase letters indicated significant differences ($P < 0.05$) in dry matter accumulation between different treatments for each organ, and different uppercase letters indicate total plant dry matter accumulation, with the LSD multiple range test. CK, control treatment; SV6, spraying EDAH at V6; SV12, spraying EDAH at V12; TS, twice-spraying EDAH; $D_{4.5}$, planting density of 4.5 × $10^4$ plants $ha^{-1}$; $D_{5.25}$, planting density of 5.25 × $10^4$ plants $ha^{-1}$; $D_{8.25}$, planting density of 8.25 × $10^4$ plants $ha^{-1}$.

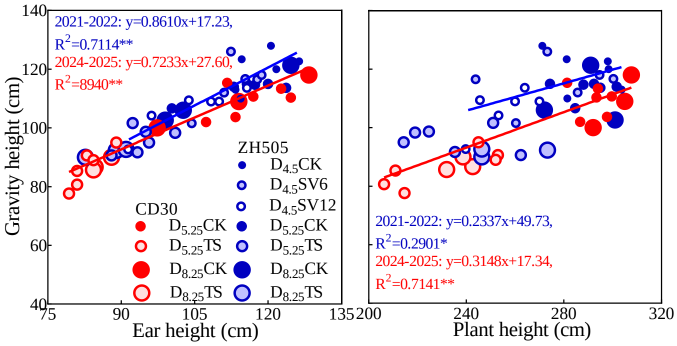


**Fig. S3** Plant height and ear height in relation to gravity height in 2021-2022 and 2024-2025. * and ** represent significant differences at $P < 0.05$ and $P < 0.01$, respectively. The red and blue solid lines represent the data fits for 2024-2025 and 2021-2022, respectively. CK, control treatment; SV6, spraying EDAH at V6; SV12, spraying EDAH at V12; TS, twice-spraying EDAH; $D_{4.5}$, planting density of 4.5 × $10^4$ plants $ha^{-1}$; $D_{5.25}$, planting density of 5.25 × $10^4$ plants $ha^{-1}$; $D_{8.25}$, planting density of 8.25 × $10^4$ plants $ha^{-1}$.

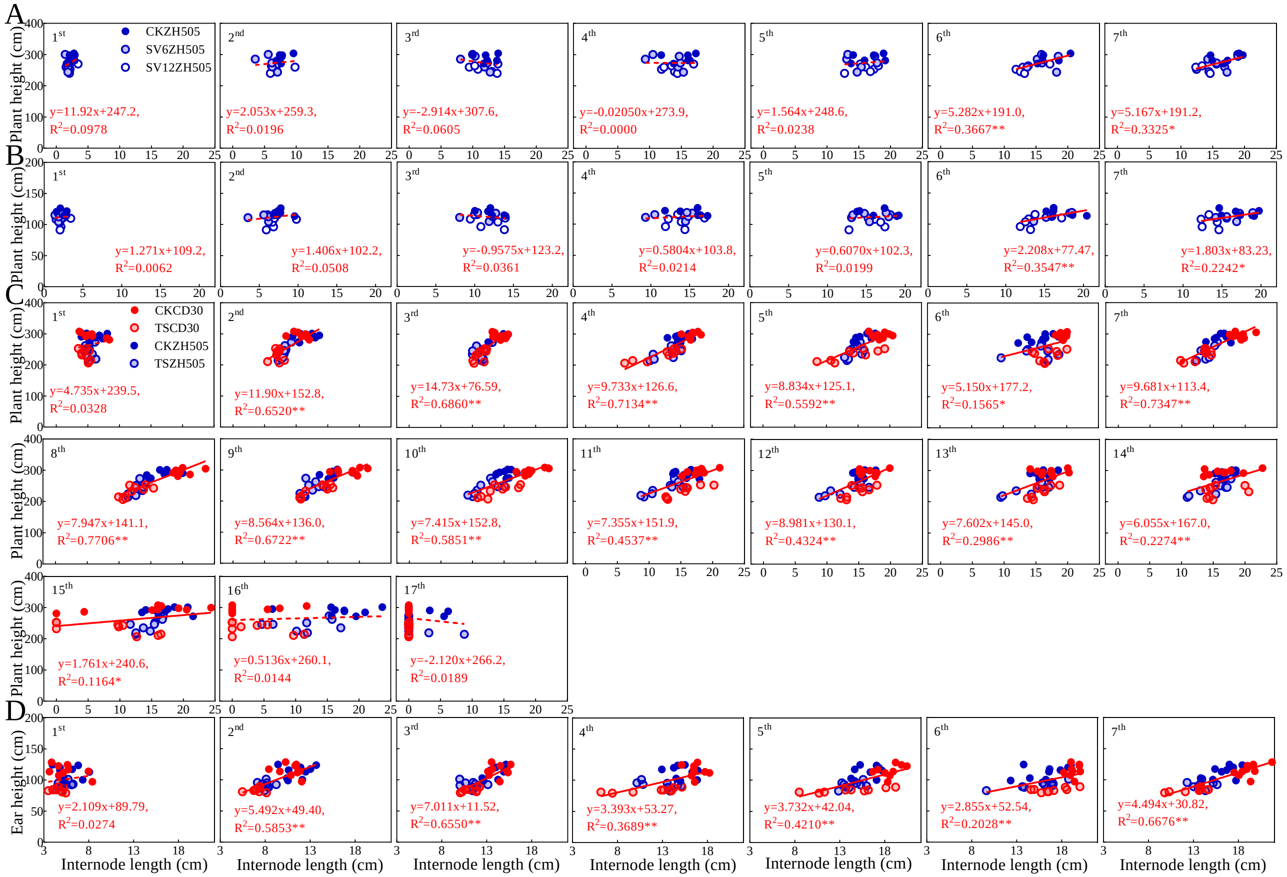


**Fig. S4** The relationship between different internode lengths and plant height as well as ear height in 2021-2022 (A and B) and 2024-2025 (C and D). Solid lines indicate a significant or highly significant linear relationship, while dashed lines indicate that the linear relationship is not significant. * and ** indicate that correlation is significant at the $P < 0.05$ level and $P < 0.01$ level, respectively, and no markings indicate that correlation is not significant. CK, control treatment; SV6, spraying EDAH at V6; SV12, spraying EDAH at V12; TS, twice-spraying EDAH.

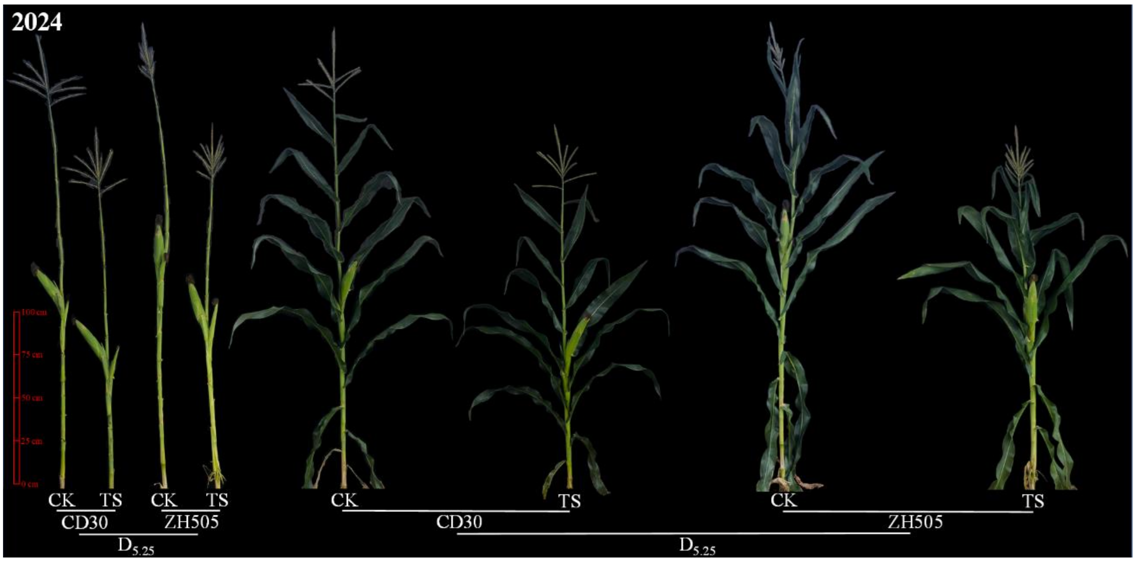


**Fig. S5** The plant morphology affected by twice-spraying plant growth regulator EDAH at 5.25 × $10^4$ plants $ha^{-1}$ planting density in 2024. CK, control treatment; TS, twice-spraying EDAH; $D_{5.25}$, planting density of 5.25 × $10^4$ plants $ha^{-1}$.

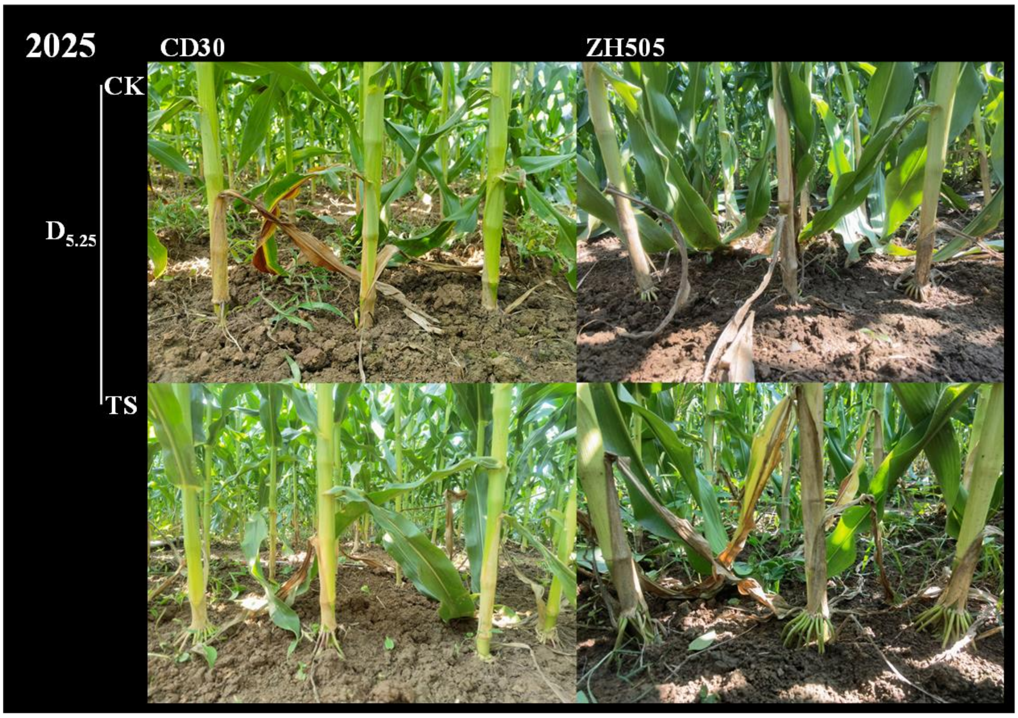


**Fig. S6** The root traits affected by twice-spraying plant growth regulator EDAH at 5.25 × $10^4$ plants $ha^{-1}$ planting density in 2025. CK, control treatment; TS, twice-spraying EDAH; $D_{5.25}$, planting density of 5.25 × $10^4$ plants $ha^{-1}$.
